# Evaluating the Effect of Supported Systematic Work Environment Management During the COVID-19 Pandemic: Protocol for a Mixed Methods Study

**DOI:** 10.2196/34152

**Published:** 2022-03-10

**Authors:** Patrik Haraldsson, Axel Ros, Dirk Jonker, Kristina Areskoug Josefsson

**Affiliations:** 1 School of Health and Welfare Jönköping Academy for Improvement of Health and Welfare Jönköping University Jönköping Sweden; 2 Occupational Safety and Health Care Region Jönköping County Jönköping Sweden; 3 Futurum–Academy for Healthcare Region Jönköping County Jönköping Sweden; 4 Faculty of Health Studies VID Specialized University Sandnes Norway; 5 Department of Behavioural Science Oslo Metropolitan University Oslo Norway

**Keywords:** occupational health interventions, implementation, mixed methods, COVID-19 pandemic, COVID-19, pandemic, occupational health, health interventions, health care, support services, employee health

## Abstract

**Background:**

The work environment is a complex phenomenon in which many factors interact. Scientific research indicates a relation between the work environment and employee health, staff turnover, patient satisfaction, and patient safety. There is a great need for knowledge on how to conduct work environment interventions and practical work environment management to maximize benefits to the employees.

**Objective:**

The aim of this study is to explore how Occupational Health Service (OHS) support will affect the work environment, sick leave, staff turnover, patient satisfaction, and patient safety during and following the COVID-19 pandemic in a medical ward setting.

**Methods:**

A mixed methods evaluation of a concurrent work environment quality improvement project at the Department of Internal Medicine and Geriatrics in a local hospital in the south of Sweden will be performed.

**Results:**

The mixed methods evaluation of the quality improvement project received funding from Futurum–Academy for Health and Care, Jönköping County Council and Region Jönköping County, and the study protocol was approved by the Swedish Ethical Review Authority. The work environment quality improvement project will continue between May 2020 and December 2021.

**Conclusions:**

The study might contribute to increased knowledge of how work environment interventions and practical work environment management can impact the work environment, and employee health, staff turnover, patient satisfaction, and patient safety. There is a need for knowledge in this area for OHS management to provide increased benefits to employees, employers, and society as a whole.

**International Registered Report Identifier (IRRID):**

DERR1-10.2196/34152

## Introduction

### Work Environment

“Work environment” is a broad term that implies everything workers are exposed to when working. The work environment can relate to work tools; air, noise, and light; psychological aspects; work organization; and well-being at work [1].

The work environment can be affected by both internal work environment factors as well as external societal factors [2]. Internal work environment aspects must be considered to understand the complexity of the work environment and its effects on human health [3-5]. The physical, environmental, and organizational/social aspects of the work environment must be understood separately [3], and the interaction between these work environment factors must be considered to understand the work environment. The complexity of internal work environment factors is exemplified in a meta-analysis showing associations among workload, job control, decision authority, and social support at work and chronic low back pain [5]. The exposure of work environment factors must be understood in relation to its exposure intensity, duration, and frequency. This is exemplified by a meta-analysis evaluating the effects on lower back pain, showing that both intensity and frequency are important aspects of the exposure [4]. External societal factors such as income inequality, social trust, and public health must also be taken into consideration, since these affect the work environment and occupational health [2].

The COVID-19 pandemic is another example of an external societal factor affecting the work environment and occupational health. Problems related to the work environment, such as high demands and a low degree of support at work, were present in the health care sector before the pandemic [6]. Research shows a high degree of mental health problems such as stress, depression, and sleep disorders in health care personnel as a consequence of the pandemic [7].

Scientific research shows conflicting results regarding work environment interventions and the effect on human health, with some studies showing no effect [8-11] and other studies showing positive effects [12-14]. In addition, there is a lack of both quantitative and qualitative evidence about what type of interventions will most improve the resilience and mental health of frontline workers during epidemics and pandemics [15]. Research to improve knowledge in this area is a high priority, since scientific evidence supports a link between the work environment and health [4,16], staff turnover [17,18], patient satisfaction [19], and patient safety outcomes [20].

### Improvement Science

Improvement science might be a suitable approach to deal with the complexity of the work environment [21]. Improvement science has been defined as “a data-driven change process that aims to systematically design, test, implement, and scale change toward systemic improvement, as informed and defined by the experience and knowledge of subject matter experts” [22]. Two central parts of improvement science are the implementation of actions and ongoing evaluations during the actions [22].

An improvement science approach is somewhat supported by a recent systematic review of interventions to improve the work environment in health care, showing that a participatory approach, continuous ongoing improvement projects, and tailoring interventions to the workplace needs are important aspects of interventions to improve nurses’ work environments [23].

### Systematic Work Environment Management

These previous results support the use of systematic work environment management (SWEM) and Occupational Health Service (OHS) support in SWEM. SWEM is a provision from the Swedish Work Environment Authority that describes mandatory work by the employer to minimize ill health and accidents at work, and promote a satisfactory working environment [24]. SWEM is conducted through risk assessment, measures, and follow-up as a continuous process. When competence within the employer’s own activity is insufficient for SWEM, the OHS can be contacted for support [24].

The OHS has a key role in supporting the health and work ability of employees in many settings [25]. The OHS has been endorsed by both the World Health Organization and the International Labor Offices as a means of ensuring a safer, healthier, happier, and more productive workforce [26]. The OHS is characterized as experts who can deliver high-quality services aimed at the working environment, and that are not offered by the employers [25]. Requirements for effective collaboration between employers and the OHS include flexible long-term contracts, effective collaboration with shared goals, frequent contact, trust, and the OHS strategically shifting from being curative to preventive [25]. The SWEM provisions include a requirement for employers without adequate internal competence to contact the OHS for support in the process. However, this is rarely enforced by labor inspectors since they find that the OHS often lacks competence in SWEM [27].

### Structured Multidisciplinary Work Evaluation Tool

The Structured Multidisciplinary Work Evaluation Tool (SMET) was developed through action research [28], as no method for OHS support in SWEM was found. SMET consists of four parts, performed in continuous iterations: (1) start-up discussions with the workplace, (2) risk assessment, (3) tailored measures, and (4) evaluation.

The risk assessment has two parts. The first part is the SMET questionnaire by which the employees evaluate the work environment. The second part is an objective in-depth analysis of the workplace, performed by the OHS. The SMET questionnaire consists of 30 items divided into three domains: physically demanding factors, environmentally demanding factors, and psychosocially demanding factors. Each domain consists of a few self-estimating items where the employees rate the degree of work-related problems on a 1-10 scale. Each domain also has one item where the employee will rate which of the previous self-estimating items constitutes the worst problem, and finally an open-ended item. The SMET questionnaire has been evaluated regarding content validity [28], intrarater reliability in the analysis of the open-ended items, and test-retest reliability of the self-estimating items [29], and has been shown to reflect the true physical workload of nursing assistants in a medical ward setting [30]. The objective in-depth analysis of the workplace is a deeper evaluation of the result in the SMET questionnaire, and entails visiting the workplace and, for example, measuring noise levels and narrow spaces or collecting sick leave data [28,29].

It is of great importance to increase the knowledge of how OHS support, with SMET in SWEM, should be conducted to benefit employers and employees, and what types of benefits can be achieved.

The aim of this study is to explore how OHS support, with SMET in SWEM, will affect the work environment, sick leave, staff turnover, patient satisfaction, and patient safety during and following the COVID-19 pandemic in a medical ward setting.

## Methods

### Design

The study involves an action research approach, with evaluation of a concurrent work environment quality improvement project using an interactive mixed methods design [31].

### Sample

Ongoing evaluation is conducted with all employees at the Department of Internal Medicine and Geriatrics in a local hospital in the south of Sweden based on a work environment project at the department. Involved professions are specialist physicians, resident physicians, intern physicians, nurses, nursing assistants, and care administrators. Inclusion criteria are individuals working at the studied department who want to participate in the research project. Exclusion criteria are hourly employees and employees on sick leave and parental leave.

### Clinical Intervention

The work environment project started at the Department of Internal Medicine and Geriatrics in May 2020 to promote the work environment and health during and following the COVID-19 pandemic. Mapping and reporting the work environment will be conducted in three steps at the department, every quarter from June 2020 to December 2021: (1) mapping the work environment with a questionnaire to all employees, (2) meeting with a reference group, and (3) meeting with the management.

The reference group consists of six employees, from different parts of the department and with different professions. Participants in the reference group were based on a pragmatic sample according to willingness to participate.

Results regarding the work environment from the questionnaire and the meeting with the reference group are compiled and presented to the management. Based on the results, the management can initiate tailored interventions in the work environment. Due to the COVID-19 pandemic, no objective in-depth analysis in the actual workplace is conducted, since this would have meant additional staff being present physically at the department.

### Data Collection

Research data will be collected quarterly through interactive acquisition and evaluation of qualitative and quantitative data regarding the work environment, leadership qualities, and work environment interventions within the work environment project. The work environment quality improvement project started in May 2020. Quantitative data will be collected with the SMET questionnaire for evaluation of the work environment, and with three questions regarding leadership qualities from the Copenhagen Psychosocial Questionnaire 3 (COPSOQ III). Both questionnaires have been tested and have shown good psychometric properties [28,29,32,33]. The use of the three items of leadership quality in COPSOQ III in isolation is supported by good internal consistency and floor/ceiling effects in these items [33].

Qualitative data will be collected through meetings with a reference group and manager interviews. Meetings with the reference group, lasting approximately 1.5 hours, will be held every quarter and will be led by the first author. The results from the questionnaires will be presented to the reference group, who will then discuss the result based on three reflection topics: *Is the result correct? Causes? Solutions?* The task of the reference group is to support the OHS in interpreting the results based on their work context. The results from the reference group will be compiled using written meeting diaries (by the first author), with these topics.

Monthly interviews will be conducted by the first author with the Deputy Head of the department for continuous evaluation of occupancy rate, ongoing work environment interventions, and other organizational interventions at the department. The interviews will be conducted by telephone and will last 20-30 minutes. The results from these interviews will be compiled using written meeting diaries (by the first author) with the topics occupancy rate, ongoing work environment interventions, and other organizational interventions at the department.

Data on sick leave and staff turnover will be collected from the human resources organization, from January 2015 to the end of the project in December 2021. Data on patient satisfaction will be collected from national registers at the Swedish Association of Local Authorities and Regions, and data on patient safety will be collected from the national quality register Senior Alert.

### Data Analysis

The Department of Internal Medicine and Geriatrics has approximately 240 employees. Power analysis showed that to identify a moderate effect size (Cohen W>0.30) in the SMET questionnaire with an α value of .05, power of 0.80, and 2 degrees of freedom, a sample of at least 54 individuals/measurement opportunities is needed. Qualitative data in the SMET questionnaire will be analyzed by content analysis, as described in Haraldsson et al [29]. Qualitative data from the monthly interviews with the Deputy Head of the department and from meetings with the reference group will be compiled using written meeting diaries (by the first author). The use of research notes such as written meeting diaries has been shown to offer data of good quality, being less time-consuming and more cost-efficient than verbatim transcription of interviews [34]. Data on sick leave, staff turnover, patient satisfaction, and patient safety will be collected and divided into two parts: the time before the COVID-19 pandemic (January 2015 to February 2020), and during and following the COVID-19 pandemic (March 2020 to December 2021) for comparison with a reference group consisting of several other departments of internal medicine. A mixed methods approach, with integration of both quantitative and qualitative data, will be used to increase the understanding of the results [31].

### Ethical Considerations

This study is a mixed methods intervention study, where the interactive research follows an ongoing quality improvement project. Questionnaire data on the work environment and leadership might be considered sensitive personal data, and data on sick leave are definitely considered as such. The collected data might be regarded as sensitive concerning personal integrity and will therefore be presented only at the group level. Conducting a study at only one department in a hospital might have the risk that an individual employee will be indirectly identified. This risk is considered insignificant since the department has 240 employees. Email addresses of all the employees will be acquired from the department. The questionnaires will be sent by the online questionnaire program esMaker. The questionnaires will be anonymized in esMaker. This means that the anonymization will be conducted by the esMaker system and not by the researchers. The results from the reference group will be compiled at the group level, which implies that these data will be anonymized as well.

Workplace data on sick leave and staff turnover will be used, as these data are continuously collected as routine practice by the regions in Sweden. Regarding data on sick leave and staff turnover, the personal integrity will be secured by obtaining the data at the group level by the human resource department. No individual data will be acquired, handled, or presented by the research group. The collection of these data is important to be able to achieve the scientific aims, and to evaluate if the quality project was successful in promoting the work environment and health.

Informed written consent will be collected from the head of the department and from the participants in the reference group meetings before the study is started. All employees at the involved department will receive oral and written information about the follow-up research project. Informed consent will be collected from all 240 employees, through the questionnaire, regarding the work environment, but it will not be collected regarding data on sick leave and staff turnover, since these data will only be presented at the group level and collection of such is part of an ongoing quality project at the clinic. Data from the questionnaire, diaries from the reference group, sick leave, staff turnover, and diaries from the monthly interviews with the Deputy Head of the department will be stored on a safe hard drive in Region Jönköping County in accordance with General Data Protection Regulation. No individual data, only data at the group level, will be stored. The study protocol was developed in accordance with the Helsinki Declaration [35] and the Swedish Ethical Review Act [36], and was approved by the Ethical Review Authority on October 26, 2020 (Dnr: 2020-03891).

## Results

The mixed methods evaluation of the quality improvement project received funding from Futurum–Academy for Health and Care, Jönköping County Council and Region Jönköping County, and the study protocol was approved by the Swedish Ethical Review Authority. The work environment quality improvement project started at the Department of Internal Medicine and Geriatrics in May 2020. Mapping and reporting the work environment to the management have been conducted every quarter from June 2020 to September 2021. On the basis of the results, the management has conducted tailored interventions to promote the work environment and employee health continuously during and following the COVID-19 pandemic. A scientific evaluation of the work environment quality improvement project will continue when the project ends in December 2021.

## Discussion

### Principal Results

There is great complexity in assessing a work environment. The conflicting results in work environment intervention studies and the suboptimal use of OHS in SWEM might be an outcome of this complexity. The results from this study will contribute to bridging the knowledge gap between SWEM and effective interventions in this field. A deeper understanding of factors linked to practical work environment management in hospitals can benefit employee health, staff turnover, patient satisfaction, and patient safety.

Central parts of SWEM and improvement science are ongoing evaluations and implementation of actions. Considering ongoing evaluations, valid and reliable tools for measurement are central to increasing the understanding and learning in the process. Our previous research has shown the SMET questionnaire to be a valid and reliable method for evaluation of the work environment [28,29], which ensures good quality of the risk assessment in SMET.

The study is a mixed methods evaluation of a concurrent work environment quality improvement project conducted before, during, and following the COVID-19 pandemic. The COVID-19 pandemic has shown that there is a great need for increased knowledge about how to protect health care personnel, regarding work environment stress, during disease epidemics and pandemics [7]. A recent Cochrane report showed a lack of both quantitative and qualitative evidence with regard to how resilience and mental health can be increased in frontline workers during and after epidemics and pandemics. The authors state that research to determine the effectiveness of such interventions is a high priority [15].

The results from this study will be used to improve the work environment in the regional context but will also contribute to knowledge in work environment interventions from a wider perspective. The results will be disseminated through national and international conferences as well as scientific journals.

### Conclusions

The study might add knowledge about work environment management and intervention studies with SMET, and how to conduct work environment interventions with a systems approach, a topic in great need of increased knowledge [37]. The study might also contribute to increased knowledge of how work environment interventions and practical work environment management can impact other factors linked to the work environment. Increased knowledge in this area is of great importance, since scientific research indicates a relation between the work environment and employee health [38-40], staff turnover [17,18], patient satisfaction [19], and patient safety [18,20]. There is a need for knowledge in this area for OHS management to increasingly benefit employees, employers, and society as a whole.

## Abbreviations

COPSOQ III: Copenhagen Psychosocial Questionnaire 3
OHS: Occupational Health Service
SMET: Structured Multidisciplinary Work Evaluation Tool
SWEM: systematic work environment management
